# From Inattention to Impulsivity: The Relationship Between Distinct Symptom Dimensions of ADHD and Eating Disorder Symptoms

**DOI:** 10.1192/j.eurpsy.2026.10445

**Published:** 2026-07-31

**Authors:** B. Kardaş, S. G. Evinç

**Affiliations:** 1 Psychology, Ankara Science University; 2 Child and Adolescent Psychiatry and Mental Health, Hacettepe University, Ankara, Türkiye

## Abstract

**Introduction:**

Attention-deficit/hyperactivity disorder (ADHD) has been increasingly linked to disordered eating behaviors, yet the contribution of specific symptom dimensions of ADHD remains unclear. Understanding whether inattention and hyperactivity/impulsivity predict distinct eating disorder (ED) symptom clusters may help clarify shared mechanisms and inform targeted interventions.

**Objectives:**

Eating disorders are inherently multidimensional, involving not only maladaptive eating behaviors such as bingeing, purging, and dietary restriction but also ongoing concerns with body weight and appearance. Considering this complexity, the present study examines the role of ADHD symptom dimensions in predicting specific clusters of eating disorder symptoms, including binge–purge behaviors, weight/shape concerns, and restrictive eating patterns.

**Methods:**

Participants were 389 adults aged 18–58 years (*M* = 26, *SD* = 7.97). ADHD symptoms were measured with the Adult ADHD Self-Report Scale. Eating disorder symptoms were assessed using the Munich Eating and Feeding Disorder Questionnaire and the Eating Disorder Examination Questionnaire, providing total and subscale scores. Four hierarchical regression analyses were conducted, using total scores of ED, binge–purge behaviors, weight/shape concerns, and restrictive eating as the outcome variables. Gender, age, and body mass index (BMI) were entered in the first step as control variables, and ADHD symptom dimensions were added in the second step as predictors.

**Results:**

ADHD symptoms significantly predicted the total scores of ED beyond demographic variables, accounting for an additional 15% of the variance (*ΔR²* = .15, *p* < .001). Both inattention (*β* = .21, *p* < .001) and hyperactivity/impulsivity (*β* = .25, *p* < .001) were significant positive predictors. For binge–purge behaviors, inattention (*β* = .21, *p* < .001) and hyperactivity/impulsivity (*β* = .28, *p* < .001) again emerged as strong predictors, with the model explaining 47% of the variance. For weight/shape concerns, only hyperactivity/impulsivity (*β* = .15, *p* = .004) was significant, explaining 33% of the variance. Similarly, for restrictive eating, hyperactivity/impulsivity (*β* = .15, *p* = .008) was the only predictor, with the model accounting for 21% of the variance. Across all models, female gender, younger age, and higher BMI consistently predicted higher ED scores.

**Conclusions:**

ADHD dimensions exhibit distinct patterns of association with ED symptom clusters. Accordingly, while both inattention and hyperactivity/impulsivity relate to total scores of ED and binge–purge behaviors, only hyperactivity/impulsivity predicts weight/shape concerns and restrictive eating. These findings highlight the importance of considering specific ADHD symptom dimensions in understanding and addressing disordered eating behaviors.

**Disclosure of Interest:**

None Declared

